# MKRN1 regulates the expression profiles and transcription factor activity in HeLa cells inhibition suppresses cervical cancer cell progression

**DOI:** 10.1038/s41598-024-56830-8

**Published:** 2024-03-13

**Authors:** Xiang Dong, Yuling Zhan, Suwan Li, Minghui Yang, Yu Gao

**Affiliations:** 1 School of Life Science, Bengbu Medical College, No. 2600 Donghai Road, Bengbu, 233030 Anhui China; 2 Research Center of Clinical Laboratory Science, School of Laboratory Medicine, Bengbu Medical College, Bengbu, 233030 Anhui China; 3 School of Basic Courses, Bengbu Medical College, Bengbu, 233030 Anhui China; 4 Laboratory Animal Center, Bengbu Medical College, Bengbu, 233030 Anhui China; 5 Anhui Province Key Laboratory of Translational Cancer Research, Bengbu Medical College, Bengbu, 233030 Anhui China

**Keywords:** Cancer, Cell biology, Cancer

## Abstract

Cervical cancer is one of the most common gynecologic malignancies worldwide, necessitating the identification of novel biomarkers and therapeutic targets. This study aimed to investigate the significance of MKRN1 in cervical cancer and explore its potential as a diagnostic marker and therapeutic target. The results indicated that MKRN1 expression was up-regulated in cervical cancer tissues and correlated with advanced tumor stage, higher grade, and poor patient survival. Functional studies demonstrated that targeting MKRN1 effectively inhibited cell proliferation, migration, and invasion, highlighting its critical role in tumor progression and metastasis. Moreover, the knockdown of MKRN1 resulted in altered expression patterns of six transcription factor-encoding genes, revealing its involvement in gene regulation. Co-expression network analysis unveiled complex regulatory mechanisms underlying the effects of MKRN1 knockdown on gene expression. Furthermore, the results suggested that MKRN1 might serve as a diagnostic marker for personalized treatment strategies and a therapeutic target to inhibit tumor growth, metastasis, and overcome drug resistance. The development of MKRN1-targeted interventions might hold promise for advancing personalized medicine approaches in cervical cancer treatment. Further research is warranted to validate these findings, elucidate underlying mechanisms, and translate these insights into improved management and outcomes for cervical cancer patients.

## Introduction

Cervical cancer (CESC) is one of the most common gynecologic malignancies worldwide, accounting for a significant number of cancer-related deaths in women^1^. It arises from transforming cervical epithelial cells due to persistent infection with high-risk human papillomavirus (HPV) types^2^. Despite advances in screening programs and the availability of prophylactic HPV vaccination, the incidence and mortality rates of cervical cancer remain alarming in certain regions, particularly in low-resource settings where access to healthcare services is limited^3^.

The molecular mechanisms underlying cervical cancer development and progression are complex and multifactorial. Numerous genetic and epigenetic alterations have been identified in cervical cancer, including aberrant expression of oncogenes and tumor suppressor genes^4^, dysregulation of signaling pathways^5^, and disruption of cellular processes such as proliferation^6^, apoptosis^7^, and DNA repair^8^. Elucidating the key molecular players involved in cervical carcinogenesis is crucial for developing effective strategies for early detection, risk assessment, and targeted therapies^9^.

The makorin ring finger protein 1 (MKRN1) gene belongs to the makorin ring zinc finger protein family, which is characterized by a conserved ring finger domain^10^. The gene is located on human chromosome 7, contains eight exons and seven introns, and its full cDNA length is 1449 nt. MKRN1 has been implicated in diverse biological processes, including ubiquitin-mediated protein degradation, mRNA splicing, and regulation of gene expression^11^. Emerging evidence suggests that MKRN1 may function as an oncogene or tumor suppressor, depending on the context and cancer type. For instance, in breast cancer, MKRN1 has been reported to promote tumor growth and metastasis through its involvement in epithelial-mesenchymal transition and cell cycle regulation^12^. The previous reports suggested that MKRN1 might be a useful adjunct biomarker in liquid-based cervical cytology screening^13^, and it might play an important role as an E3 ligase in the occurrence and development of cervical cancer^14^.

Based on the potential role of MKRN1 in cancer development and progression, it is imperative to explore its expression patterns and functional significance in cervical cancer. Although previous studies have investigated the involvement of MKRN1 in various cancer types, limited information is available on its contribution to cervical carcinogenesis. Therefore, in this study, we aimed to investigate the expression levels and biological implications of MKRN1 in cervical cancer using a combination of bioinformatics analysis and in vitro experiments. By elucidating the role of MKRN1 in cervical cancer, we hope to uncover novel insights into the molecular mechanisms underlying this disease and identify potential therapeutic targets for intervention.

## Results

### MKRN1 was highly expressed in cervical cancer based on bioinformatics analysis

**Figure 1:**
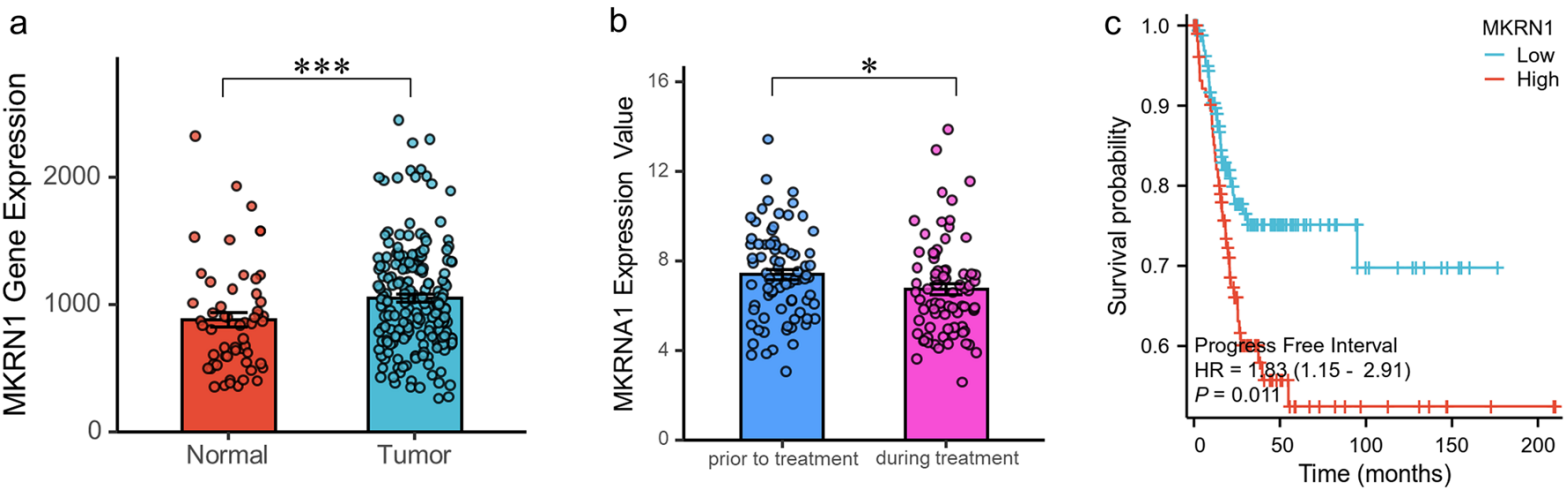
Differential expression and survival analysis of MKRN1 gene in CESC. (**a**) The MKRN1 expression levels in normal cervical tissue samples (n = 56) and non-paired cervical cancer samples (n = 189). (**b**) The MKRN1 expression levels in cervical cancer samples prior to treatment and during treatment of the CESC patients (n = 39). (**c**) The CESC patients with high MKRN1 mRNA levels in CESC tumors, compared to those with low MKRN1 mRNA levels, had a significantly poorer progression-free interval (PFI) survival probability.

To investigate the correlation between the expression level of MKRN1 and the progression of cervical cancer, a comprehensive bioinformatics analysis was conducted using publicly available databases. The mRNA expression data of MKRN1 in both cervical tumor tissues and normal tissues were retrieved from the TNMplot web platform. The analysis revealed a significant upregulation of MKRN1 expression in non-paired cervical cancer samples (n = 189) compared to normal cervical samples (n = 56) (*P* = 0.00231; Fig. 1a). In the GSE3578 dataset, MKRN1 expression levels were found to be significantly down-regulated in CESC patients (n = 39) who received radiotherapy alone or radiotherapy plus concomitant chemotherapy (*P* = 0.0225; Fig. 1b). Moreover, a cohort of 306 CESC patients from The Cancer Genome Atlas (TCGA) datasets exhibited a significantly lower progression-free interval (PFI) survival probability in the high MKRN1 expression group compared to the low expression group (Fig. 1c).

### Down-regulation of MKRN1 could suppress HeLa cell proliferation

**Figure 2:**
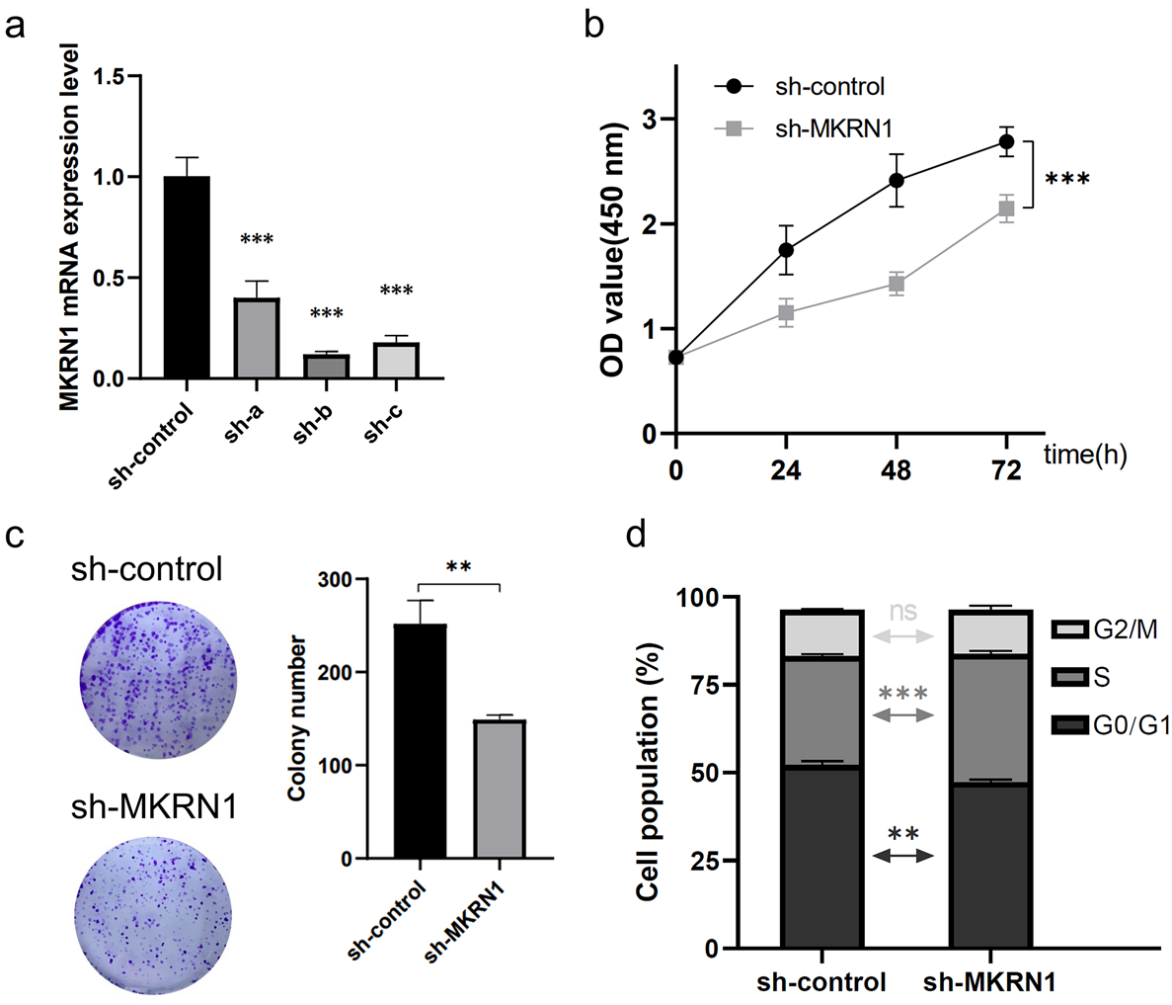
Effects of MKRN1 knockdown on cell proliferation of HeLa cells. (**a**) MKRN1 knockdown by transfection with empty vector (sh-control) or with MKRN1 shRNA (sh-a, sh-b, and sh-c) was validated by qRT-PCR. (**b**) The effect of MKRN1 interference on HeLa cells was detected by a CCK8 assay. (**c**) The proliferation effect of MKRN1 interference in colony formation assay. (**d**) The cell cycle distribution of sh-control/sh-MKRN1 on HeLa cells.

The impact of MKRN1 knockdown on cell proliferation in HeLa cells was assessed through the use of shRNA. The effectiveness of MKRN1 knockdown was confirmed by performing qRT-PCR analysis (Fig. 2a). The data demonstrated that sh-MKRN1-b (sh-b) exhibited the highest efficiency in suppressing MKRN1 gene expression, resulting in up to an 89% reduction compared to the control group (sh-control). Subsequent experiments were conducted using sh-b (named as sh-MKRN1). The results obtained from a CCK8 assay indicated a significant decrease in the proliferation ability of the sh-MKRN1 group compared to the control group at 24 h, 48 h, and 72 h time points (Fig. 2b). Furthermore, the colony formation assay revealed a notable decline of cell proliferation in the sh-MKRN1 group when compared with the control group (Fig. 2c). To gain further insights into the effects of MKRN1 on HeLa cell proliferation, flow cytometry was employed to examine changes in the cell cycle. The findings demonstrated that MKRN1 knockdown impeded the transition of HeLa cells from the G0/G1 phase to the S phase (Fig. 2d). These results collectively suggested that MKRN1 might play a crucial role in regulating the proliferation of HeLa cells.

### Down-regulation of MKRN1 could suppress HeLa cell migration and invasion

**Figure 3:**
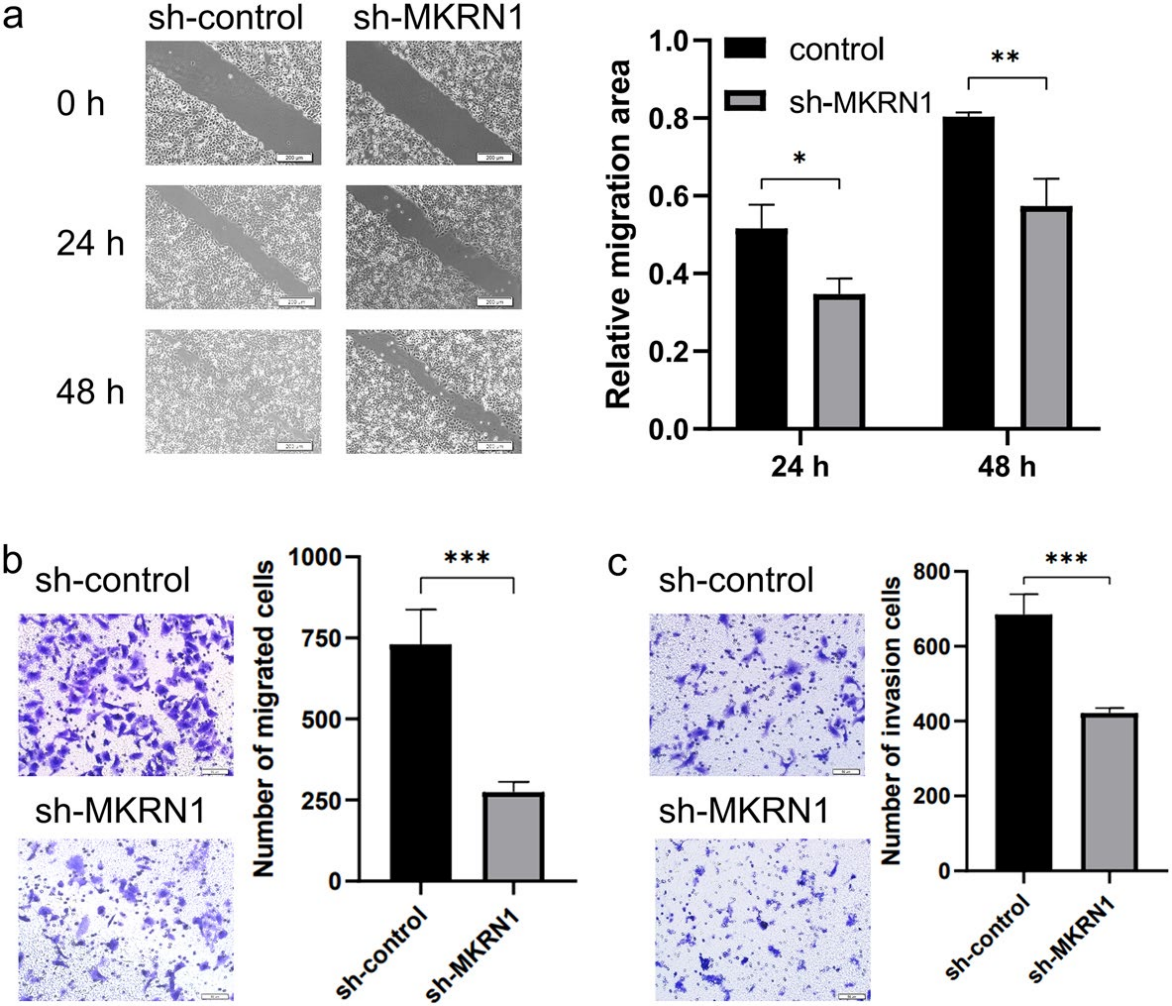
Down-regulation of MKRN1 inhibited HeLa cell migration and invasion. (**a**) Scratch assay to detect HeLa cell migration ability. (**b**) Transwell assay to detect HeLa cell migration ability. (**c**) Transwell assay (containing matrix gel) to detect HeLa cell invasion ability.

In this study, the migration and invasion of HeLa cells were assessed through cell scratch and Transwell experiments. The results of the scratch assay indicated that migration in HeLa cells was significantly impaired when MKRN1 expression was down-regulated (sh-MKRN1), as compared to cells in the control group (sh-control), at 24 h and 48 h time points (Fig. 3a). Similarly, in the Transwell migration assay, a significant reduction in migrating HeLa cells was observed upon the knockdown of MKRN1 (Fig. 3b). Additionally, the Transwell invasion assay revealed a reduction in the number of HeLa cells passing through the Matrigel upon the down-regulation of MKRN1 (Fig. 3c). Taken together, these findings suggested that the migration and invasion capabilities of HeLa cells could be influenced by the expression of MKRN1.

### Down-regulation of MKNR1 changed the gene expression profiles of Hela cell

**Figure 4:**
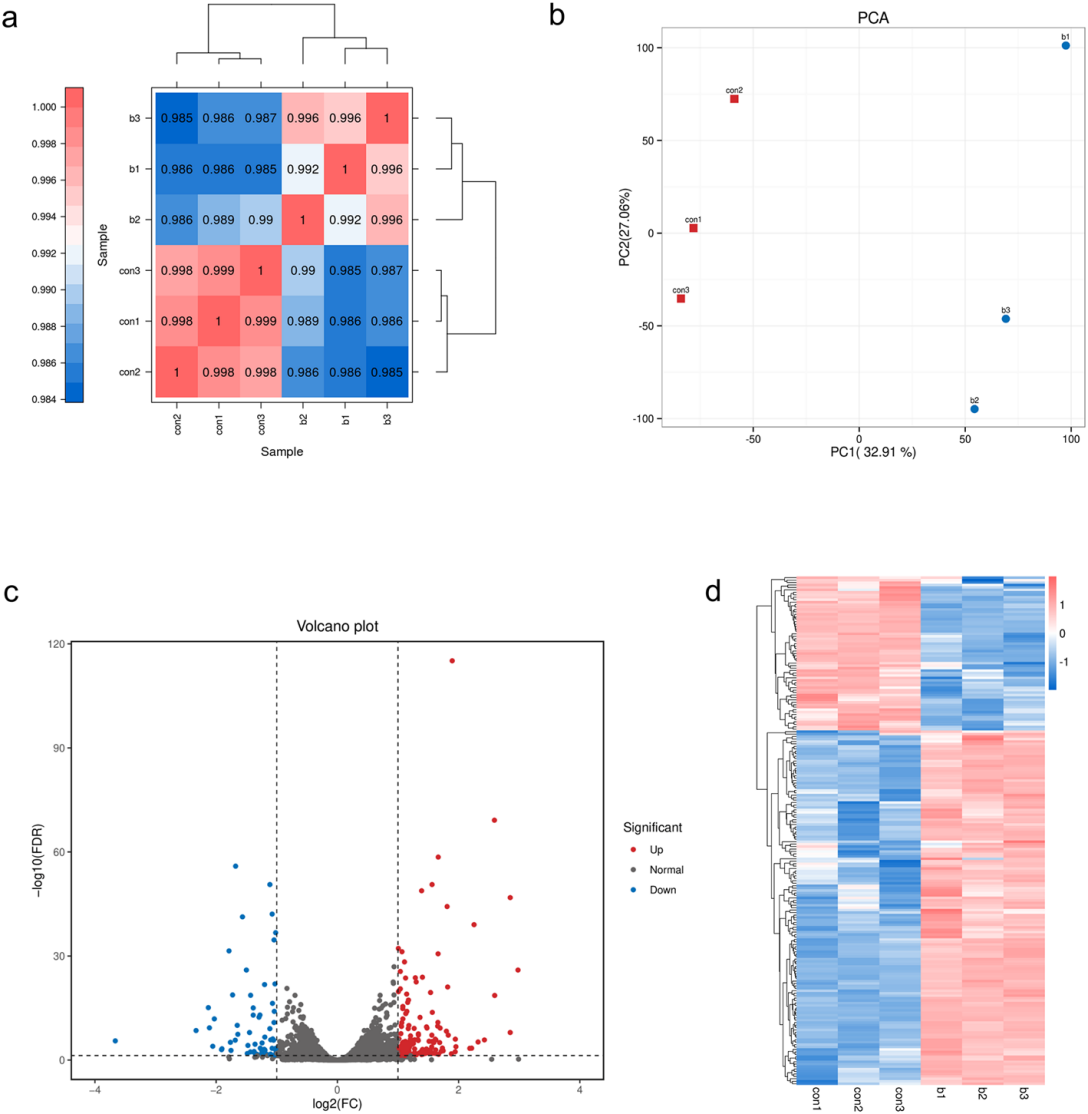
Down-regulation of MKNR1 could change the gene expression profiles of HeLa cells. (**a**) The correlation heatmap between MKNR1 knockdown (b1, b2, and b3) and control (con1, con2, and con3) samples. (**b**) Principal component analysis (PCA) plot. (**c**) Differentially expressed genes between the sh-MKRN1 group and the sh-control group. (**d**) Hierarchical cluster analysis of differentially expressed genes between the sh-MKRN1 group (b1, b2, and b3) and the sh-control group (con1, con2, and con3).

To elucidate the role of MKNR1 in HeLa cells, an RNA-seq analysis was conducted to examine the expression patterns of the shRNA knockdown group (sh-MKRN1) and the control group (sh-control) of HeLa cells. The data files for RNA-seq have been deposited in the Sequence Read Archive (SRA) database under PRJNA987347. Despite having only three biological replicates in each group, a significant correlation between the sh-MKRN1 group and the sh-control group was observed (Fig. 4a). The result of principal component analysis (PCA) also showed samples were divided into two groups (Fig. 4b). The analysis of differential gene expression identified genes with |log2 (Fold Change)| > 1 and adjusted *p* < 0.05 as differentially expressed genes (DEGs). Among these, a total of 208 DEGs were induced by the shRNA knockdown of MKRN1, consisting of 145 up-regulated genes and 63 down-regulated genes (Fig. 4c). The complete list of DEGs can be found in Supplementary Table S1. Furthermore, hierarchical clustering analysis was performed on the differentially expressed genes, grouping genes with similar expression patterns together (Fig. 4d).

### Overview of affected biological function by the shRNA knockdown of MKRN1

**Figure 5:**
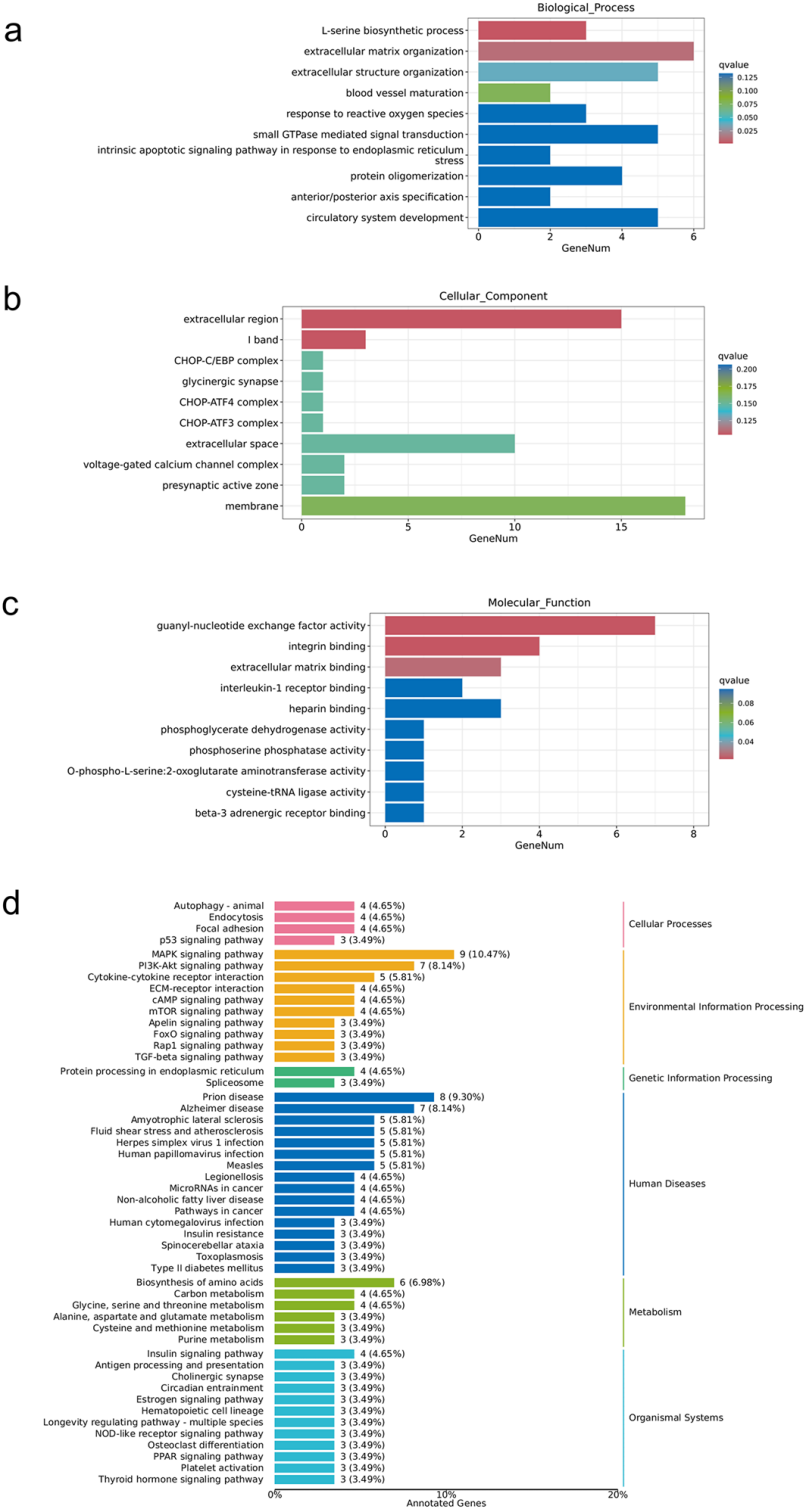
The potential biological functions of differentially expressed genes. (**a**) The enriched GO biological processes terms. (**b**) The enriched GO cellular component terms. (**c**) The enriched GO molecular function terms. (**d**) Kyoto Encyclopedia of Genes and Genomes (KEGG) pathway enrichment.

To investigate the potential biological functions of MKRN1-related genes, GO enrichment analyses and KEGG pathway annotation analyses were performed on the significantly differentially expressed genes. The top ten of enriched GO biological processes of the regulated genes were mainly related to the L-serine biosynthetic process, extracellular matrix organization, extracellular structure organization, and so on (Fig. 5a). The enriched GO cellular component terms were related to the I band and extracellular region, and so on (Fig. 5b). The enriched GO molecular function terms were primarily associated with integrin binding, guanyl-nucleotide exchange factor activity, extracellular matrix binding, and so on (Fig. 5c). The results of KEGG pathway enrichment showed that DGEs were involved in pathways associated with cellular processes, environmental information processing, genetic information processing, metabolism, and so on (Fig. 5d).

### Analysis of potential MKRN1‑regulated transcription factor and enrichment biological function analysis

To gain a deeper understanding of the regulatory mechanisms involved in the MKRN1 knockdown, transcription factors (TFs) of DEGs were identified and annotated based on the Ami-nalTFDB database in this study. A total of 6 DEGs encoding TFs were identified with the shRNA knockdown of MKRN1, and these TFs belonged to 5 TF families (Table 1). Transcription factor activity was evaluated by using CoRegNet package. The co-operated TF interactions were listed in the Supplementary Table S2. To further investigate the regulatory networks between these identified TFs and their targeted genes, the h-Lincorn model in conjunction with CoRegNet to was used to construct a co-regulatory network. The co-expression network of transcription factors was shown in the following Fig. 6a. And the hierarchical clustering heatmap on TF activity was shown in the Fig. 6b.

**Table 1:** The annotation of transcription factors of differentially expressed genes.

#Gene_ID	Symbol	log2FC	Regulated	Family
ENSG00000182759	MAFA	1.196	Up	TF_bZIP
ENSG00000009950	MLXIPL	1.695	Up	bHLH
ENSG00000157514	TSC22D3	1.125	Up	TSC22
ENSG00000204514	ZNF814	− 1.245	Down	zf-C2H2
ENSG00000175197	DDIT3	1.053	Up	TF_bZIP
ENSG00000164749	HNF4G	1.268	Up	RXR-like

**Figure 6:**
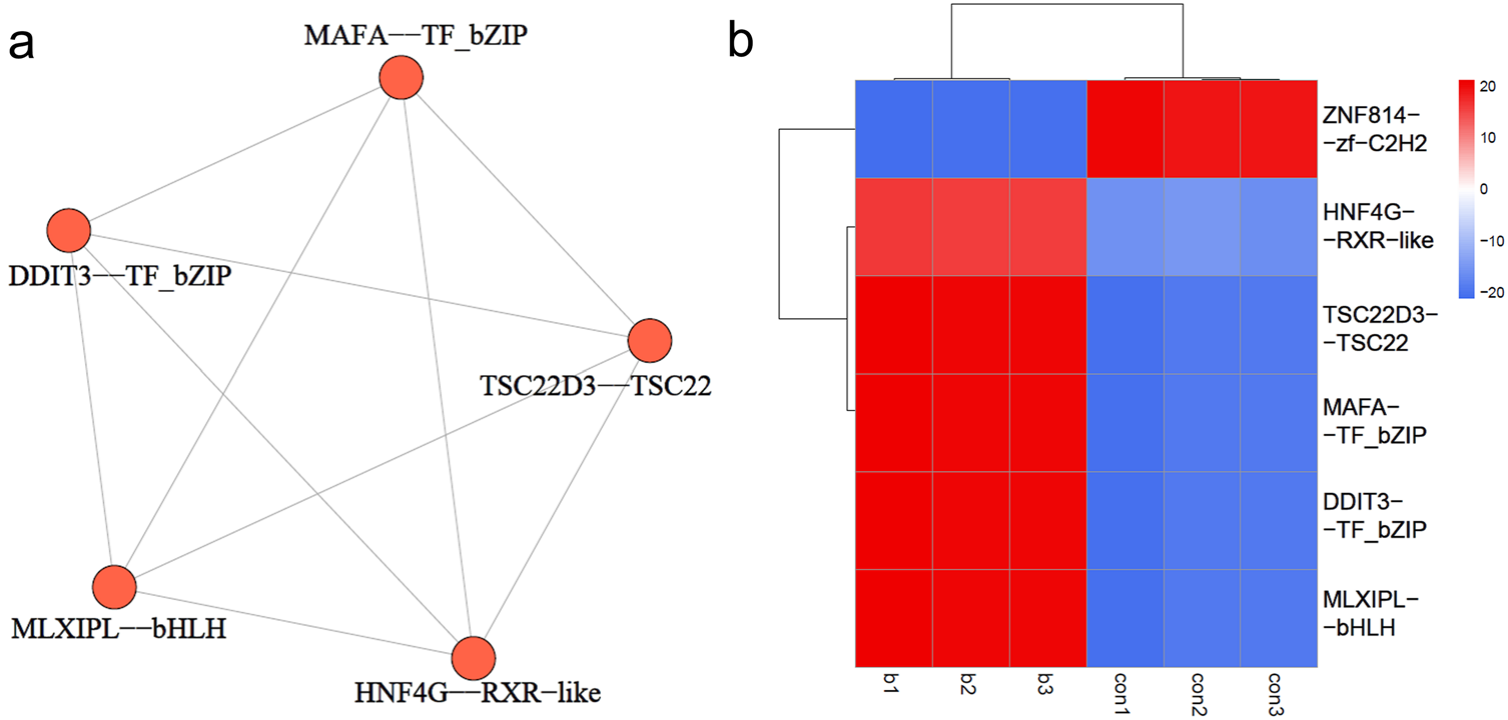
Transcription Factor Activity Analysis. (**a**) Co-operation network of transcription factors. Nodes represent transcription factors (TFs). Edges represent co-operated relations. (**b**) Hierarchical clustering heatmap on TF activity between sh-MKRN1 group (b1, b2, and b3) and the sh-control group (con1, con2, and con3). Each column represents a sample, and each row represents a TF. The red ones represented the TFs with positive regulating effects on genes, and the blue ones represented that of negative ones.

## Discussion

In this study, a comprehensive bioinformatics analysis was conducted to investigate the correlation between MKRN1 expression and the progression of cervical cancer. A significant upregulation of MKRN1 expression in non-paired cervical cancer samples compared to normal cervical tissue samples was observed, suggesting a crucial role of MKRN1 in the development and progression of cervical cancer. Improved treatment response was associated with down-regulation of MKRN1 expression in CESC patients who underwent radiotherapy alone or radiotherapy plus concomitant chemotherapy, indicating the potential of MKRN1 as a predictive marker for treatment outcomes. Furthermore, a significant association was found between high MKRN1 expression and a lower progression-free interval (PFI) survival probability in cervical cancer patients, suggesting that MKRN1 overexpression might indicate poor prognosis. These novel findings provide valuable informationinto the potential therapeutic targeting and prognostic use of MKRN1 in cervical cancer.

The results demonstrated that knockdown of MKRN1 could significantly decrease in the proliferation ability of HeLa cells, as evidenced by CCK8 and colony formation assays. Furthermore, flow cytometry analysis revealed that MKRN1 knockdown impeded the transition of HeLa cells from the G0/G1 phase to the S phase, indicating a role for MKRN1 in regulating the cell cycle. Our findings were consistent with previous studies that have identified MKRN1 as a key regulator of cell proliferation in various cancer types, such as pancreatic cancer^15^, renal clear cell carcinoma^16^, and colorectal cancer^17^. Further research is needed to elucidate the molecular mechanisms underlying the role of MKRN1 in regulating cancer cell migration and invasion.

The results of RNA-seq analysis showed that the knockdown of MKRN1 led to significant changes in gene expression profiles. The enriched gene ontology (GO) analyses revealed that DEGs induced by MKRN1 knockdown were associated with various biological processes, cellular components, and molecular functions. The top term of biological processes was related to regulating the L-serine biosynthetic process. Serine metabolism might play a key role in maintaining metabolic homeostasis and health^18^. And serving as a primary source of one-carbon units, serine is a significant contributor to cancer metabolism^19^ (non-melanoma skin cancer^20^, pancreatic cancer^21^, and lung cancer^22^). It has been reported that drugs, targeting serine-related one-carbon metabolic pathway, could inhibit the proliferation of cancer cells^23^, such as bladder cancer^24^, and endometrial cancer^25^. In this study, the regulation of the L-serine biosynthetic process by MKRN1 suggested that it might have a role in cell proliferation and survival. Understanding the mechanism by which MKRN1 regulates the L-serine biosynthetic process could provide valuable insights into the potential role of MKRN1 in diseases where cell proliferation and survival are deregulated.

The results of GO enrichment analysis showed that extracellular matrix organization, extracellular region, and extracellular matrix binding were listed as the top ten terms of biological processes, cellular components, and molecular functions, respectively. The extracellular matrix (ECM) is a complex network of proteins and other macromolecules that provide structural support and regulate cell behavior. Dysregulation of extracellular matrix organization can lead to various diseases, including fibrotic diseases^26^, pancreatic cancer^27^, gastric cancer^28^, breast cancer^29^, and other cancer types^30^. Furthermore, the enrichment of terms related to the extracellular region underscores the importance of these genes in regulating the cellular microenvironment. The regulation of the extracellular microenvironment is crucial for cellular proliferation, differentiation, migration, and survival. Enriching molecular functions related to extracellular matrix binding provides further evidence for the involvement of these genes in ECM regulation^31^. Extracellular matrix binding proteins interact with ECM components, mediating cell-ECM interactions and regulating ECM assembly and remodeling^32^. The findings supported previous studies that had implicated MKRN1 in the modulation of cell–matrix adhesion and signaling pathways involved in tumor progression, as well as its role in inducing the degradation of multiple oncogenic proteins and inhibiting tumor growth through the proteasomal pathway^33^. Therefore, the role of MKRN1 in extracellular matrix organization, extracellular region, and extracellular matrix binding suggested that it might play a pivotal role in maintaining the structural and functional integrity of the ECM, which is essential for tissue homeostasis and development. Additionally, KEGG pathway analysis revealed that the DEGs were involved in multiple pathways, including the MAPK signaling pathway, spliceosome, and biosynthesis of amino acids. These results were consistent with previous studies showing that MKRN1 might play a critical role in regulating cell proliferation and apoptosis through the p53 pathway^34^. Overall, the findings of this study provided new insights into the molecular mechanisms underlying the role of MKRN1 in cervical cancer progression.

In the present study, the effects of MKRN1 knockdown on gene expression and regulatory mechanisms were also investigated. The results revealed that the knockdown of MKRN1 led to altered expression of six genes encoding transcription factors, indicating that MKRN1 might play an important role in regulating transcriptional activity. It was consistent with previous studies suggesting the involvement of TFs in the regulation of cellular processes and cancer progression^35^. Furthermore, the co-expression network analysis showed that these TF-encoding genes were closely interconnected, suggesting a complex regulatory cascade that governed the effects of MKRN1 knockdown on gene expression. It provided a systems-level view of the regulatory landscape, emphasizing the complexity of transcriptional regulation in cervical cancer. Taken together, the findings might enhance our understanding of the molecular mechanisms involved in regulating gene expression by MKRN1, offering valuable implications for comprehending cancer pathogenesis and devising innovative therapeutic approaches.

There are certain limitations in our study. Firstly, this study primarily relied on bioinformatics analysis and in vitro experiments, which might not fully capture the complexity of the tumor microenvironment and the interplay between cancer cells and their surrounding tissues. Further studies using in vivo models and clinical samples would be needed to validate our findings and assess the therapeutic potential of targeting MKRN1 in a more clinically relevant context. Secondly, although a significant correlation between MKRN1 expression and clinical parameters was observed, further investigation would be required to establish the causative relationship between MKRN1 and cervical cancer progression. Thirdly, although an extensive analysis of TFs and co-regulatory networks was performed, it is important to acknowledge that other regulatory mechanisms, such as non-coding RNAs, epigenetic modifications, and post-translational modifications, might also contribute to the observed changes in gene expression. More experimental validation would be needed to confirm the functional roles of the identified TFs and their interactions. Future studies should consider conducting functional assays, such as knockdown or overexpression experiments, to validate the effects of these TFs on gene expression and cellular processes, and to provide a more complete understanding of MKRN1-mediated transcriptional regulation.

In conclusion, this study highlighted the significance of MKRN1 in cervical cancer. MKRN1 might be up-regulated in cervical cancer tissues and be associated with advanced tumor stage, higher grade, and poor patient survival. The functional studies provided evidence that targeting MKRN1 could effectively inhibit cell proliferation, migration, and invasion, highlighting its crucial role in promoting tumor progression and metastasis. Moreover, MKRN1 could potentially serve as a diagnostic marker for tailoring personalized treatment strategies and as a therapeutic target for inhibiting tumor growth, metastasis, and overcoming drug resistance in cervical cancer. However, further research is required to validate these findings, elucidate the underlying mechanisms, and translate this knowledge into improved management and outcomes for cervical cancer patients. Developing MKRN1-targeted interventions might help advance personalized medicine approaches in cervical cancer treatment.

## Methods

### Bioinformatics analysis

In this study, a comprehensive bioinformatics analysis was conducted to investigate the correlation between the expression level of MKRN1 and the progression of cervical cancer. The expression data of MKRN1 in cervical samples were obtained from three publicly accessible databases: the TNMplot web platform (https://www.tnmplot.com/), the Gene Expression Omnibus (GEO) database (https://www.ncbi.nlm.nih.gov/geo), and The Cancer Genome Atlas (TCGA) datasets (https://portal.gdc.cancer.gov). The TNMplot was a web tool for the comparison of gene expression in normal, tumor and metastatic tissues^36^. It provided expression analysis for one selected gene in one kind of tissue type using RNA-Seq based data or gene chip-based data, which included the data from paired tumor and adjacent normal tissues, or the data from non-paired tumor and normal tissues. For CESC analysis, there were only mRNA expression data of MKRN1 in non-paired cervical tumor tissues (n = 189) and normal cervical tissues (n = 56) from chip-based studies in the TNMplot database. A Mann–Whitney U test was used to compare the difference of MKRN1 between non-paired cervical tumor and normal cervical tissues. The dataset of GSE3578^37^, which included the cervical squamous cell carcinoma patients who received radiotherapy alone or radiotherapy plus concomitant chemotherapy, was retrieved from. The expression levels of MKRN1 were analyzed to evaluate the correlation with different treatment regimens in CESC patients (n = 39). In the TCGA database, a total of 306 RNA-seq data from CESC patients with clinical records were available. However, there were only three samples of adjacent non-cancerous tissues of CESC. Because of the low numbers of adjacent non-cancerous tissue samples, the comparison of paired normal and adjacent cervical tumors was not analyzed in this study. The expression levels of MKRN1 in the cohort of 306 CESC patients from were obtained, and the patients were divided into two groups based on high and low MKRN1 expression levels. Kaplan–Meier survival analysis and log-rank test were conducted to assess the differences in progression-free interval (PFI) survival probability between the high and low MKRN1 expression groups. The data used in this study were obtained from the publicly available datasets. Therefor, ethical approval was not required.

### Cell culture and lentivirus transfection

The human cervical cancer cell line HeLa was purchased from the cell bank of the Chinese Academy of Sciences in Shanghai and was cultured in appropriate growth media supplemented with fetal bovine serum and antibiotics were cultured in DMEM (Dulbecco's Modified Eagle Medium) (Procell, Wuhan, China) with 10% Gibco™ fetal bovine serum (Invitrogen, AUS), 1% Penicillin–Streptomycin (10,000 U/mL) (Invitrogen, USA), at 37 °C in a 5% CO_2_ humidified atmosphere. Cells were maintained under standard cell culture conditions. For the knockdown of MKRN1 expression, lentiviral particles encoding MKRN1-specific shRNA sequences were generated and used to transduce HeLa cells. The target sequence of sh-a was 5′-GCTAACTACAAAGTCATCCCT-3′, the target sequence of sh-b was 5′-GCAATTTGAGAGCAAGATCAT-3′, and the target sequence of sh-c was 5-GCGAGATGTTGCTTATGCTTT-3′. The empty vehicle was used as a control. The transfection flow was performed according to the reagent manufacturer's instructions using the Lipofectamine™ 2000 Transfection Reagent (Invitrogen, USA). The cells were selected with 1.5 μg/mL puromycin for two weeks. The efficiency of MKRN1 knockdown was validated by quantitative real-time polymerase chain reaction (qRT-PCR). GAPDH was used as the internal reference gene. The following primers were used for expression analysis: MKRN1-fw: 5′-CATGGGGTTTGTAAGGAAGGAG-3′. MKRN1-rv: 5′-GCACACTACACTATACGGACTGT-3′; GAPDH-fw: 5′-ATGGGTGTGAACCATGAGAAGTA-3′; GAPDH-rv: 5′-GAGTGGGTGTCGCTGTTGAAGTC-3′. Each sample was run in triplicate by qRT-PCR (Applied Biosystems™ 7500, USA). Relative gene expression level was measured by 2^−ΔΔCt^ relative quantitative method.

### Cell counting kit 8 (CCK8) assay

A cell counting kit 8 (CCK8) (Beyotime Biotechnology, China) assay was performed to assess the effect of MKRN1 knockdown on cell proliferation. The cells from the sh-MKRN1 group and the control group were plated in a 100 μL culture medium containing 1 × 10^3^ into 96-well plates for cell culture 24, 48, and 72 h. Then, 10 μL of CCK8 reagent was added to each well. After incubated at 37 °C for 2 h, the absorbance at 450 nm (OD) of each well was measured.

### Colony formation assay

A colony formation assay was conducted to investigate the effect of MKRN1 knockdown on cell proliferation. The cells from the sh-MKRN1 group and control group were seeded into culture plates at a low density (1 × 10^3^ cells/well into 6-well plates), and were allowed to grow for 10 days. After that, the colonies were fixed in 4% paraformaldehyde, stained with 0.5% crystal violet, and gently rinsed three times with PBS. A spot with more than 50 cells was counted as one clone.

### Cell cycle assay

To analyze changes in the cell cycle, the cells from two groups were harvested by trypsinization, washed with phosphate-buffered saline (PBS), and fixed in ice-cold 70% ethanol. Fixed cells were washed with PBS and stained with propidium iodide (PI) solution. After incubation at 37 °C for 30 min in a light-proof environment, flow cytometry analysis was performed using a flow cytometer (Backman Coulter, USA).

### Cell scratch assay

For the scratch assay, the cells were seeded onto 6-well culture plates and allowed to grow until they reached approximately 90% confluency. A scratch was created on the cell monolayer using a sterile 200 μL pipette tip, ensuring consistent gap width and length. The plates were then gently washed with PBS to remove detached cells or debris. Fresh growth media was added to the wells to facilitate cell migration. The scratch area was imaged immediately after creating the scratch (0 h) and at the time points of 24 h and 48 h. The images were captured at the same location in each well to ensure consistency.

### Cell migration and invasion assays

The impact of MKRN1 down-regulation on cell migration and invasion was evaluated using Transwell migration and invasion assays. The cells were seeded onto Transwell chambers (Costar, USA) with or without Matrigel (BD Biosciences) coating and incubated for 24 h. The cells that migrated or invaded through the membrane were fixed in 4% paraformaldehyde, stained with 0.5% crystal violet, and counted under a microscope. The number of invasive cells was counted in five random fields of view.

### RNA sequencing

The RNA samples of the cells from the sh-MKRN1 group and the control group were subjected to sequencing analysis with three replicates per group at Biomarker Technologies Co., LTD (Beijing, China). Before library construction, the purity, concentration, and integrity of the RNA samples were assessed using NanoDrop and Agilent 2100. RNA samples that met the quality standards were further processed for library preparation. The resulting high-quality libraries were pooled according to pre-designed target data volume and subsequently subjected to sequencing on the Illumina NovaSeq6000 platform. Raw data underwent stringent filtering to obtain clean data with optimal quality. Subsequently, the clean data were aligned to the human reference genome employing the HISAT2 software^38^. The data files for RNA-seq have been deposited in the Sequence Read Archive (SRA) database under PRJNA987347.

### Screening of differentially expressed genes

After processing and analyzing the RNA sequencing data, the alignment results were transferred to the program StringTie for transcript assembly^39^. The gene expression levels of RNA-seq were expressed as FPKM (Fragments Per Kilobase of transcript sequence per Millions base pairs sequenced). The differentially expressed genes were identified using the package of DESeq algorithm in the R software. The resulting p-values were adjusted using Benjamini and Hochberg’s approach for controlling the false discovery rate. The thresholds to screen differentially expressed genes (DEGs) were |log2Fold change (FC)| > 1 and adjust *p* value < 0.05.

### Gene function enrichment analysis

To predict the potential functions of differentially expressed genes between the sh-MKRN1 group and the control group, gene ontology (GO) analysis and Kyoto Encyclopedia of Genes and Genomes (KEGG) functional pathway analysis were employed. The GO analysis was used to annotate genes based on biological processes, molecular functions, and cellular components. The KEGG pathway analysis was utilized to identify significant pathways that were enriched with differentially expressed genes. Enrichment was considered significant when the adjusted *p* value was less than 0.05.

### Transcription factor analysis

To identify potential transcription factors (TFs) associated with the differentially expressed genes, the gene sets were selected as candidate genes and subjected to prediction using the AnimalTFDB database via HMMsearch^40^. The activity of the identified TFs was assessed using the CoRegNet R package^41^. Subsequently, a co-regulatory network between the identified TFs and their targeted genes was constructed based on the h-Lincorn model using CoRegNet.

### Statistical analysis

All the above analyses were carried out by using R software version 4.0.3. All experiments were repeated three times, and data were expressed as mean ± standard deviation. Differences between the two groups were analyzed using independent samples t-test and paired t-test. Correlation analysis was based on Pearson's correlation test. Statically significance was defined as *p* < 0.05 (*), and *p* < 0.01 (**).

## Conmpeting interests

The authors declare no competing interests.

### Supplementary Information

Supplementary Information.

## Data Availability

The authors confirm that the data supporting the findings of this study are available within the article and its supplementary materials. The raw sequence data of RNA were deposited in the NCBI SRA database with the accession number of PRJNA987347 (https://www.ncbi.nlm.nih.gov/bioproject/PRJNA987347). Publicly available datasets were analyzed in this study, that can be found in the TNMplot web platform (https://www.tnmplot.com/), GEO database (https://www.ncbi.nlm.nih.gov/geo), and The Cancer Genome Atlas (https://portal.gdc.cancer.gov).
