# Supplementary material for: MKRN1 regulates the expression profiles and transcription factor activity in HeLa cells inhibition suppresses cervical cancer cell progression
Source: Sci Rep. 2024 Mar 13;14:6129. doi: 10.1038/s41598-024-56830-8 (PMC10937657; doi:10.1038/s41598-024-56830-8)
Supplement: Supplementary file 1 — Supplementary Information. [file 41598_2024_56830_MOESM1_ESM.pdf]

Title page

MKRN1 regulates the expression profiles and transcription factor activity in HeLa cells inhibition suppresses cervical cancer cell progression

Xiang Dong[1,2], Yuling Zhan[1,2], Suwan Li[1,2], Minghui Yang[2,3], Yu Gao[1,4,5*]

[1] School of Life Science, Bengbu Medical College, Bengbu, Anhui 233030, China

[2] Research Center of Clinical Laboratory Science, School of Laboratory Medicine, Bengbu Medical College, Bengbu, Anhui 233030, China

[3] School of Basic Courses, Bengbu Medical College, Bengbu, Anhui 233030, China

[4] Laboratory Animal Center, Bengbu Medical College, Bengbu, Anhui 233030, China

[5] Anhui Province Key Laboratory of Translational Cancer Research, Bengbu Medical College, Bengbu, Anhui 233030, China

Corresponding Author: * Yu Gao, School of Life Science, Laboratory Animal Center, Anhui Province Key Laboratory of Translational Cancer Research, Bengbu Medical College, No. 2600 Donghai Road, Bengbu, 233030, China. Tel: +86-19105521190. Fax: +86-552-3175396. Email: gaoyu@bbmc.edu.cn

Supporting information

Supplemental Table S1. The list of differentially expressed genes (DEGs)

**Symbol**	con1_FPKM	con2_FPKM	con3_FPKM	b1_FPKM	b2_FPKM	b3_FPKM	FDR	log2FC	regulated
CTGF	17.97	17.47	19.03	5.94	5.44	6.07	1.25E-56	-1.68	down
CYR61	125.81	120.48	126.73	58.56	59.28	59.11	2.47E-51	-1.11	down
HSPA8	1033.94	994.74	1070.43	466.42	489.98	525.52	8.08E-43	-1.08	down
THBS1	4.10	4.16	3.75	1.52	1.34	1.30	5.11E-42	-1.57	down
HSPA1B	100.34	96.37	102.24	49.62	52.02	49.78	1.87E-37	-1.02	down
HSPA1A	104.19	101.06	108.59	50.08	53.93	52.05	2.32E-35	-1.04	down
CPA4	8.32	7.77	7.37	2.80	2.09	2.07	3.39E-32	-1.79	down
MATN2	4.19	4.34	4.45	1.54	1.70	1.64	1.11E-26	-1.50	down
UPK3B	15.76	17.08	16.89	7.48	7.65	8.53	1.10E-22	-1.03	down
PCDH7	3.83	3.93	3.73	1.83	1.67	1.62	1.71E-22	-1.20	down
VWF	1.09	0.94	1.01	0.29	0.33	0.32	1.67E-19	-1.73	down
OLFML1	4.19	4.65	4.73	1.93	1.67	1.65	2.09E-19	-1.43	down
DKK1	13.85	13.15	15.11	6.62	7.11	6.82	4.44E-17	-1.07	down
ANKRD1	3.84	4.07	3.19	1.20	0.63	0.78	7.68E-16	-2.13	down
PSG4	7.88	8.42	7.04	2.53	3.72	2.84	9.23E-16	-1.39	down
CD3EAP	8.09	8.94	9.49	4.79	4.09	3.73	9.22E-15	-1.04	down
SEMA7A	3.79	3.37	3.00	1.52	1.32	1.29	1.02E-13	-1.28	down
SPOCK1	4.01	3.79	3.07	1.43	1.13	1.68	1.10E-13	-1.39	down
NRXN3	1.56	2.41	2.14	1.02	0.89	1.11	4.33E-13	-1.29	down
AC005324.3	1.72	2.50	3.89	0.75	0.57	0.73	1.34E-12	-2.03	down
IGFN1	2.09	2.75	2.12	1.65	1.06	1.11	1.46E-11	-1.05	down
PRICKLE1	1.61	1.39	1.58	0.26	0.33	0.36	9.72E-11	-1.65	down

**Symbol**	con1_FPKM	con2_FPKM	con3_FPKM	b1_FPKM	b2_FPKM	b3_FPKM	FDR	log2FC	regulated
MDGA1	0.30	0.30	0.29	0.07	0.05	0.08	5.10E-10	-2.11	down
Homo_sapiens_newGene_5697	2.06	2.36	2.31	1.31	1.09	1.07	8.82E-10	-1.11	down
KCND3	0.79	0.76	0.61	0.06	0.13	0.21	3.09E-09	-2.33	down
BMP4	3.32	2.42	3.35	1.14	1.11	1.07	1.13E-08	-1.45	down
MYOCD	0.57	0.69	0.79	0.28	0.20	0.14	3.74E-08	-1.64	down
KLHDC7A	1.24	1.32	1.70	0.63	0.67	0.59	2.36E-07	-1.20	down
COBLL1	1.30	2.37	1.38	0.73	0.50	0.68	7.99E-07	-1.07	down
TRIM54	3.08	3.04	3.43	1.30	1.52	1.71	1.48E-06	-1.10	down
Homo_sapiens_newGene_3705	1.18	1.29	1.23	0.64	0.40	0.26	1.87E-06	-1.68	down
PLEKHG4B	1.33	0.70	0.93	0.47	0.34	0.50	1.94E-06	-1.05	down
RUBCNL	0.31	0.25	0.27	0.03	0.00	0.03	2.94E-06	-3.66	down
PSG5	1.34	1.16	1.03	0.22	0.38	0.38	7.56E-06	-1.76	down
FAM222A	1.34	1.36	1.93	0.68	0.58	0.74	1.16E-05	-1.24	down
GPC5	1.22	1.12	0.98	0.47	0.53	0.50	2.71E-05	-1.36	down
Homo_sapiens_newGene_1325	0.87	0.94	1.01	0.45	0.32	0.26	5.98E-05	-1.48	down
Homo_sapiens_newGene_3623	6.34	6.11	2.47	0.61	1.41	1.55	1.06E-04	-2.06	down
CDH5	1.04	1.28	1.22	0.31	0.75	0.55	3.74E-04	-1.27	down
PSAPL1	0.98	1.20	1.33	0.58	0.46	0.74	3.93E-04	-1.01	down
Homo_sapiens_newGene_7254	2.93	2.36	2.56	1.53	1.18	1.46	4.07E-04	-1.08	down
PSG9	1.03	1.00	0.47	0.21	0.32	0.15	5.50E-04	-1.90	down
ARHGDIB	3.54	3.63	4.35	1.61	1.86	2.14	6.35E-04	-1.03	down
SPTB	0.42	0.69	0.84	0.23	0.19	0.33	7.69E-04	-1.21	down
PIWIL3	0.38	0.37	0.29	0.06	0.13	0.08	1.01E-03	-1.91	down
Homo_sapiens_newGene_4286	1.10	1.41	1.00	0.45	0.42	0.65	1.12E-03	-1.20	down
Homo_sapiens_newGene_4056	3.38	3.38	4.69	2.24	2.19	1.97	1.46E-03	-1.19	down
GPR78	0.76	0.38	0.32	0.10	0.14	0.19	1.69E-03	-1.76	down
ZNF814	1.76	2.19	2.25	1.32	1.40	1.46	2.13E-03	-1.24	down
TMED7-TICAM2	1.19	1.63	1.12	0.85	0.50	0.23	3.42E-03	-1.37	down

**Symbol**	con1_FPKM	con2_FPKM	con3_FPKM	b1_FPKM	b2_FPKM	b3_FPKM	FDR	log2FC	regulated
PGBD5	0.15	0.15	0.11	0.05	0.06	0.04	4.45E-03	-1.43	down
Homo_sapiens_newGene_3123	1.60	2.44	2.48	0.74	0.88	0.92	5.27E-03	-1.38	down
ITGB3	0.94	0.86	0.59	0.30	0.54	0.33	7.50E-03	-1.01	down
Homo_sapiens_newGene_849	0.94	1.46	1.12	0.57	0.35	0.55	9.33E-03	-1.25	down
AP000944.2	7.88	3.55	9.43	2.06	2.28	3.30	1.31E-02	-1.49	down
MAMDC2	0.54	0.27	0.33	0.14	0.16	0.15	1.60E-02	-1.35	down
CPA2	0.92	0.70	1.48	0.37	0.48	0.35	1.99E-02	-1.35	down
Homo_sapiens_newGene_5602	0.93	1.09	0.75	0.42	0.38	0.61	2.03E-02	-1.10	down
CD101	0.34	0.43	0.34	0.19	0.17	0.12	2.16E-02	-1.24	down
DOK3	0.86	1.14	1.29	0.74	0.27	0.25	2.19E-02	-1.33	down
CLSTN2	0.19	0.13	0.20	0.09	0.11	0.05	2.20E-02	-1.11	down
AMIGO3	0.80	0.63	1.14	0.56	0.22	0.41	2.27E-02	-1.17	down
RASGRP1	0.70	0.58	0.46	0.55	0.17	0.26	3.34E-02	-1.04	down
ASNS	22.87	22.28	20.61	83.56	85.16	86.98	7.65E-116	1.89	up
CHAC1	2.41	2.46	2.34	14.05	15.05	16.80	7.00E-70	2.59	up
PCK2	8.66	8.66	7.58	27.16	27.32	28.43	3.00E-59	1.66	up
SESN2	3.29	3.31	3.20	10.25	9.61	9.87	2.47E-51	1.56	up
DDIT4	60.78	59.94	65.22	154.08	173.51	172.07	1.53E-49	1.39	up
ADM2	0.40	0.30	0.42	2.79	2.68	2.90	1.45E-47	2.85	up
FZD10	3.14	3.76	2.90	11.11	12.23	11.97	5.40E-45	1.81	up
Homo_sapiens_newGene_1429	0.58	0.47	0.57	2.94	2.48	2.52	9.19E-40	2.25	up
TXNIP	41.71	37.76	41.56	83.34	83.40	82.60	6.46E-33	1.01	up
CARS	28.15	25.80	28.37	56.21	60.55	60.71	5.76E-32	1.07	up
PHGDH	2.40	2.76	2.63	8.03	9.64	8.65	2.30E-31	1.66	up
PSAT1	55.19	52.17	60.03	118.19	132.25	121.86	5.00E-29	1.11	up
Homo_sapiens_newGene_6595	1.43	1.23	1.16	11.12	10.36	10.19	1.11E-26	2.98	up
USH1C	24.72	23.31	25.90	49.95	53.66	54.55	2.82E-26	1.04	up
Homo_sapiens_newGene_6037	1.68	1.49	1.87	5.22	4.19	4.63	1.31E-24	1.40	up

**Symbol**	con1_FPKM	con2_FPKM	con3_FPKM	b1_FPKM	b2_FPKM	b3_FPKM	FDR	log2FC	regulated
C14orf180	4.84	5.05	3.91	11.04	11.19	11.19	1.87E-24	1.29	up
TSC22D3	7.19	6.32	6.24	14.61	14.47	14.31	2.23E-24	1.12	up
KIF21B	1.22	1.81	1.62	3.74	3.27	3.24	2.98E-23	1.30	up
CBS	2.44	2.20	3.24	8.47	9.28	11.34	8.53E-22	1.82	up
PSPH	11.97	10.91	12.33	23.91	29.78	27.35	2.91E-21	1.04	up
SMOC1	10.84	10.05	11.26	21.01	23.08	22.24	1.87E-20	1.01	up
Homo_sapiens_newGene_6038	1.65	1.13	1.53	4.54	4.03	4.35	3.34E-20	1.54	up
GPT2	5.27	5.02	4.36	10.06	11.15	11.98	9.57E-20	1.14	up
PPFIA4	0.22	0.12	0.19	1.65	1.01	1.10	2.35E-19	2.59	up
NOTCH3	1.04	1.02	0.94	2.37	2.11	2.45	4.91E-18	1.17	up
SPINK5	9.19	10.81	8.89	26.38	19.20	20.53	2.38E-17	1.16	up
ADSSL1	6.43	6.16	6.37	12.31	14.12	14.21	3.46E-16	1.08	up
TRIM31	4.89	4.85	4.50	9.71	11.11	10.06	1.42E-15	1.08	up
Homo_sapiens_newGene_94	1.60	1.33	1.42	3.56	3.30	3.03	9.24E-15	1.15	up
INHBE	1.49	1.66	1.37	3.85	5.08	4.58	1.76E-14	1.57	up
TVP23A	2.04	1.94	1.95	4.00	4.28	4.53	3.96E-14	1.13	up
FAM167A	0.84	0.89	0.76	2.18	2.18	2.01	5.00E-13	1.36	up
Homo_sapiens_newGene_389	3.55	3.99	3.43	8.78	7.73	7.07	1.34E-12	1.08	up
NUPR1	1.31	0.56	0.50	0.95	2.26	2.61	1.38E-11	1.66	up
CACNA1H	0.76	0.78	0.66	1.70	1.67	1.82	1.65E-11	1.06	up
GPCPD1	2.07	2.43	1.88	6.08	3.83	4.86	1.06E-10	1.18	up
DDIT3	10.47	8.23	11.53	22.33	21.54	20.57	1.07E-10	1.05	up
Homo_sapiens_newGene_4683	1.15	1.06	0.98	3.53	3.71	3.17	1.65E-10	1.65	up
Homo_sapiens_newGene_2984	1.60	1.83	1.73	4.03	4.12	4.23	2.04E-10	1.06	up
ECM2	1.15	1.66	1.30	4.97	3.01	3.79	2.68E-10	1.47	up
Homo_sapiens_newGene_5376	0.84	0.98	1.02	2.23	2.73	3.16	5.79E-10	1.48	up
MLXIPL	0.53	0.72	0.75	2.18	2.03	2.43	6.00E-10	1.70	up
TRIM15	1.30	1.49	1.53	3.22	3.59	3.87	7.30E-10	1.27	up

**Symbol**	con1_FPKM	con2_FPKM	con3_FPKM	b1_FPKM	b2_FPKM	b3_FPKM	FDR	log2FC	regulated
Homo_sapiens_newGene_4575	0.39	0.44	0.45	1.06	0.97	0.98	8.54E-10	1.17	up
P4HA3	0.45	0.39	0.34	1.35	1.68	1.54	4.76E-09	1.80	up
CPAMD8	0.02	0.16	0.17	0.80	1.07	1.21	1.12E-08	2.85	up
Homo_sapiens_newGene_5194	2.29	2.06	2.01	3.90	5.19	4.79	2.37E-08	1.05	up
Homo_sapiens_newGene_6043	0.45	0.37	0.47	1.19	1.00	1.13	2.76E-08	1.34	up
PRSS56	0.90	1.07	1.22	2.60	2.54	3.17	5.78E-08	1.34	up
HCAR2	0.43	0.49	0.32	1.44	1.38	1.77	6.51E-08	1.83	up
Homo_sapiens_newGene_3920	1.00	1.44	0.88	3.05	4.06	3.81	6.51E-08	1.56	up
IL20RB	1.40	1.09	1.46	3.14	3.10	3.24	1.06E-07	1.21	up
Homo_sapiens_newGene_2735	1.28	0.90	1.19	2.81	1.97	2.26	7.34E-07	1.02	up
Homo_sapiens_newGene_5032	1.40	1.09	1.12	2.48	3.06	3.12	7.99E-07	1.21	up
Homo_sapiens_newGene_106	0.29	0.28	0.21	0.82	0.87	0.87	9.39E-07	1.61	up
ADGRG3	0.19	0.28	0.33	0.84	0.99	1.31	9.43E-07	1.95	up
ERFE	1.07	1.31	1.47	2.96	2.94	3.72	1.17E-06	1.33	up
RASGEF1B	0.63	1.03	1.15	2.54	2.06	2.84	1.38E-06	1.27	up
HCAR3	0.46	0.47	0.34	1.26	1.45	1.34	1.50E-06	1.62	up
Homo_sapiens_newGene_4200	0.44	0.19	0.14	1.74	1.44	1.36	1.56E-06	2.43	up
AC007240.1	1.32	1.10	0.91	2.22	3.03	3.04	2.30E-06	1.33	up
ARHGEF6	0.16	0.29	0.15	0.60	0.53	0.55	3.80E-06	1.51	up
Homo_sapiens_newGene_5078	1.00	1.21	0.94	2.51	2.56	2.51	4.21E-06	1.12	up
Homo_sapiens_newGene_3167	0.58	0.48	0.25	1.56	1.17	1.26	4.41E-06	1.56	up
VMAC	0.89	1.14	0.80	2.46	2.10	2.11	4.57E-06	1.19	up
MASP1	0.09	0.08	0.06	0.37	0.55	0.33	6.09E-06	2.32	up
TENT5C	0.40	0.39	0.43	0.97	0.79	0.90	7.17E-06	1.09	up
GDPD1	1.10	1.71	1.16	3.38	3.19	3.76	7.39E-06	1.25	up
Homo_sapiens_newGene_4601	0.60	0.38	0.25	1.25	1.34	1.44	8.76E-06	1.59	up
GAGE12D	6.88	4.71	2.57	10.42	20.07	16.39	9.04E-06	1.69	up
Homo_sapiens_newGene_1436	2.44	1.74	2.36	4.80	4.91	5.28	1.09E-05	1.17	up

**Symbol**	con1_FPKM	con2_FPKM	con3_FPKM	b1_FPKM	b2_FPKM	b3_FPKM	FDR	log2FC	regulated
Homo_sapiens_newGene_3781	0.57	0.82	0.80	1.91	1.16	1.44	1.30E-05	1.07	up
Homo_sapiens_newGene_3360	0.63	0.46	0.54	1.17	1.29	1.58	1.61E-05	1.25	up
MAFA	0.61	0.80	0.72	1.59	1.82	1.63	2.53E-05	1.20	up
RIMS3	0.20	0.20	0.22	0.58	0.47	0.46	2.65E-05	1.23	up
ALPK1	0.45	0.43	0.54	1.32	1.09	1.01	3.26E-05	1.15	up
Homo_sapiens_newGene_6059	1.03	0.91	0.87	2.15	2.00	2.21	9.55E-05	1.09	up
AC018523.2	1.81	1.74	1.95	3.38	3.74	4.55	1.02E-04	1.05	up
ARRDC3	1.57	2.30	1.34	5.39	2.74	3.56	1.12E-04	1.12	up
TTC39B	0.17	0.19	0.13	0.37	0.41	0.39	1.24E-04	1.15	up
Homo_sapiens_newGene_6531	0.16	0.24	0.26	1.06	0.94	0.79	1.39E-04	1.96	up
Homo_sapiens_newGene_525	0.55	0.28	0.40	1.31	0.98	1.16	1.59E-04	1.56	up
GARNL3	1.24	1.07	0.92	2.25	2.44	2.48	1.83E-04	1.12	up
NT5M	1.00	0.77	1.13	2.22	2.39	2.24	2.02E-04	1.19	up
Homo_sapiens_newGene_2627	0.75	0.86	0.51	1.52	1.49	1.72	2.48E-04	1.09	up
NRTN	2.27	2.20	1.60	3.72	4.13	4.77	3.44E-04	1.02	up
Homo_sapiens_newGene_2809	0.61	0.44	0.56	0.99	1.32	1.07	3.46E-04	1.02	up
ANGPTL4	0.36	0.17	0.34	1.12	0.79	1.16	3.50E-04	1.73	up
CACNA1D	0.21	0.21	0.23	0.64	0.39	0.46	3.56E-04	1.09	up
Homo_sapiens_newGene_4821	0.10	0.12	0.17	0.69	0.63	0.55	3.75E-04	2.21	up
CA9	0.17	0.42	0.08	1.19	0.86	1.19	4.15E-04	2.18	up
DEPTOR	0.43	0.39	0.46	1.30	0.79	1.01	6.03E-04	1.27	up
PPP1R3G	0.12	0.14	0.21	0.36	0.47	0.51	6.25E-04	1.47	up
SMC1B	0.26	0.45	0.31	0.83	0.80	0.74	8.28E-04	1.15	up
Homo_sapiens_newGene_2943	0.71	1.09	1.05	3.18	2.12	2.86	8.35E-04	1.40	up
ULBP1	0.59	0.66	1.09	1.94	1.63	1.50	9.64E-04	1.07	up
Homo_sapiens_newGene_217	1.13	1.27	1.24	2.31	3.00	2.42	9.93E-04	1.04	up
GPR34	0.33	0.35	0.35	1.49	0.83	0.79	1.14E-03	1.53	up
IL1A	0.58	0.29	0.25	1.72	0.90	0.99	1.17E-03	1.62	up

**Symbol**	con1_FPKM	con2_FPKM	con3_FPKM	b1_FPKM	b2_FPKM	b3_FPKM	FDR	log2FC	regulated
Homo_sapiens_newGene_3183	0.33	0.21	0.27	0.90	0.82	0.63	1.25E-03	1.49	up
PIGZ	0.57	0.63	0.47	1.00	2.38	1.13	1.46E-03	1.15	up
Homo_sapiens_newGene_1412	0.75	1.15	1.04	2.08	2.10	2.18	1.58E-03	1.00	up
Homo_sapiens_newGene_4793	1.06	1.27	0.71	3.34	1.81	2.04	2.00E-03	1.18	up
Homo_sapiens_newGene_7223	0.23	0.13	0.29	0.79	0.59	0.94	2.08E-03	1.75	up
Homo_sapiens_newGene_5778	0.23	0.27	0.39	1.04	0.72	0.62	2.71E-03	1.39	up
Homo_sapiens_newGene_6091	0.96	0.91	0.62	2.38	0.83	1.60	2.95E-03	1.16	up
Homo_sapiens_newGene_7134	0.44	0.11	0.25	0.78	1.06	1.05	3.08E-03	1.71	up
Homo_sapiens_newGene_2628	1.02	0.74	0.53	1.28	1.75	1.72	3.35E-03	1.11	up
SLC2A4	0.27	0.40	0.47	0.85	0.71	0.95	3.92E-03	1.10	up
GNG7	0.09	0.11	0.02	0.25	0.33	0.30	4.08E-03	1.94	up
RAPGEF4	0.36	0.21	0.31	0.98	0.53	0.83	4.33E-03	1.14	up
KCNQ2	0.45	0.13	0.62	1.38	1.13	1.64	4.57E-03	1.16	up
Homo_sapiens_newGene_5539	1.17	0.95	1.05	2.47	2.13	2.47	5.09E-03	1.05	up
Homo_sapiens_newGene_4805	0.30	0.33	0.42	1.09	0.71	0.53	5.09E-03	1.12	up
HSD11B1L	0.79	1.54	0.79	1.91	1.83	2.30	5.11E-03	1.04	up
C11orf71	0.80	0.93	0.62	1.31	1.54	2.07	5.64E-03	1.10	up
GOLGA8N	0.24	0.15	0.13	0.39	0.39	0.44	6.14E-03	1.17	up
IL1B	0.72	0.43	0.60	0.93	1.93	1.60	7.11E-03	1.27	up
SLC7A11	1.89	2.98	1.87	8.68	4.02	5.04	8.03E-03	1.35	up
Homo_sapiens_newGene_910	0.33	0.37	0.52	1.06	0.89	0.72	8.03E-03	1.15	up
Homo_sapiens_newGene_2946	0.19	0.38	0.29	0.72	1.07	0.94	8.47E-03	1.50	up
Homo_sapiens_newGene_7168	1.11	0.40	0.60	1.79	1.61	2.04	9.06E-03	1.14	up
HNF4G	0.54	0.96	0.53	1.41	1.08	1.23	1.12E-02	1.27	up
SORBS2	0.04	0.06	0.36	0.27	0.16	0.24	1.16E-02	1.56	up
Homo_sapiens_newGene_421	0.25	0.16	0.11	0.59	0.56	0.55	1.17E-02	1.61	up
Homo_sapiens_newGene_7209	0.53	0.22	0.25	0.92	0.81	1.11	1.19E-02	1.45	up
Homo_sapiens_newGene_1878	0.47	0.39	0.31	0.88	0.71	0.93	1.27E-02	1.05	up

**Symbol**	con1_FPKM	con2_FPKM	con3_FPKM	b1_FPKM	b2_FPKM	b3_FPKM	FDR	log2FC	regulated
PLAC1	0.54	0.19	0.05	0.88	1.02	1.20	1.59E-02	1.88	up
Homo_sapiens_newGene_653	0.31	0.52	0.68	1.06	1.37	1.10	1.70E-02	1.05	up
Homo_sapiens_newGene_3072	0.28	0.21	0.30	0.74	0.56	0.60	1.77E-02	1.21	up
Homo_sapiens_newGene_565	0.23	0.27	0.33	0.58	0.69	0.67	1.80E-02	1.42	up
Homo_sapiens_newGene_1948	0.35	0.30	0.22	0.41	0.47	0.88	2.05E-02	1.25	up
NPW	0.81	0.46	0.18	1.67	1.25	1.61	2.16E-02	1.67	up
Homo_sapiens_newGene_3100	0.65	0.37	0.46	1.39	0.83	1.01	2.33E-02	1.06	up
Homo_sapiens_newGene_6977	0.47	0.18	0.30	1.07	0.61	0.80	2.54E-02	1.29	up
Homo_sapiens_newGene_4744	0.54	0.12	0.18	0.34	0.62	0.80	2.58E-02	1.31	up
ANG	0.41	0.91	0.30	2.21	1.11	1.17	2.61E-02	1.39	up
Homo_sapiens_newGene_873	0.71	0.19	0.19	1.04	1.69	1.13	2.84E-02	1.30	up
RASSF4	0.21	0.23	0.20	0.33	0.67	0.48	3.58E-02	1.05	up
TRIM10	0.17	0.48	0.21	0.64	0.77	0.65	4.20E-02	1.08	up
Homo_sapiens_newGene_3872	0.24	0.34	0.27	0.89	0.46	0.43	4.44E-02	1.03	up
SCUBE1	0.05	0.09	0.06	0.19	0.16	0.14	4.51E-02	1.06	up
Homo_sapiens_newGene_3149	0.45	0.24	0.41	0.85	0.83	0.86	4.72E-02	1.08	up
Homo_sapiens_newGene_5460	0.53	0.36	0.51	1.08	1.04	1.18	4.78E-02	1.08	up
Homo_sapiens_newGene_4151	0.28	0.27	0.75	0.70	1.03	1.13	4.93E-02	1.11	up

Supplemental Table S2. The list of co-operated TF interactions

Reg1	Reg2	Support	NA	nGRN	fisherTest	adjustedPvalue
DDIT3--TF_bZIP	MLXIPL--bHLH	0.13044169	821	821	7.85E-07	1.31E-06
DDIT3--TF_bZIP	MAFA--TF_bZIP	0.13044169	821	821	7.85E-07	1.31E-06
DDIT3--TF_bZIP	TSC22D3--TSC22	0.13044169	821	821	7.85E-07	1.31E-06
MAFA--TF_bZIP	MLXIPL--bHLH	0.13044169	821	821	7.85E-07	1.31E-06
MLXIPL--bHLH	TSC22D3--TSC22	0.13044169	821	821	7.85E-07	1.31E-06
MAFA--TF_bZIP	TSC22D3--TSC22	0.13044169	821	821	7.85E-07	1.31E-06
DDIT3--TF_bZIP	HNF4G--RXR-like	0.063870353	402	402	0.00028567	0.00028567
HNF4G--RXR-like	MLXIPL--bHLH	0.063870353	402	402	0.00028567	0.00028567
HNF4G--RXR-like	MAFA--TF_bZIP	0.063870353	402	402	0.00028567	0.00028567
HNF4G--RXR-like	TSC22D3--TSC22	0.063870353	402	402	0.00028567	0.00028567
